# Effects of substituting wheat by rye in diets for young fattening pigs on nutrient digestibility, performance, products of intestinal fermentation, and fecal characteristics

**DOI:** 10.3389/fvets.2023.1199505

**Published:** 2023-06-29

**Authors:** Volker Wilke, Josef Kamphues

**Affiliations:** Institute for Animal Nutrition, University of Veterinary Medicine Hannover, Hanover, Germany

**Keywords:** piglets, wheat, rye, performance, nutrient digestibility, fermentation, sustainability, fattening pigs

## Abstract

Climate change and increasing demands to reduce the environmental impact of feed production are major challenges for animal nutritionists. Compared to wheat, which is commonly used in high levels in European piglet diets, rye is more efficient in using limited resources, most importantly, water and phosphorus. As a result, its cultivation has a relatively low carbon footprint. The high amounts of non-starch polysaccharides of rye might lead to an increased intestinal fermentation with potential beneficial effects on gut health. However, the high levels of non-starch polysaccharides in rye, which have a major impact on the physico-chemical conditions of the digesta, might affect digestibility and performance especially in young animals. It was therefore of interest to compare the effects of isoenergetic diets with increasing levels of rye as a replacement for wheat fed to young fattening pigs (bodyweight: 16–40 kg). The control diet contained 69% of wheat, while in the other three experimental diets, the amount of wheat was gradually replaced (by a third in each case) with rye. Thus, the experimental diets contained 23, 46, and 69% of rye. A total of 40 young pigs were housed individually in four dietary treatment groups. During a 4 week trial, effects on performance, digestibility, products of intestinal fermentation, and fecal characteristics were evaluated. There were no negative effects on feed intake and gains, even though the feed conversion ratio increased with the highest dietary rye level (69%). Digestibility rates of organic matter and crude protein did not differ significantly. Without affecting the characteristics of the feces, numerically higher amounts of intestinal fermentation products and higher colonic digesta mass were observed.

## 1. Introduction

The choice between different cereal varieties is nowadays not only about maximum energy and protein yield per acre, but rather a solution that is best adapted to the current conditions. These are characterized by climate change (more heat- and drought-tolerant plants), environmental effects (expenditure on fertilizers and plant protection), and successes in plant breeding (yields of new varieties). In 2022, the highest annual mean temperature since weather records began was measured in Germany (1). An increase in the number of days with particularly high temperatures, but also more and longer phases without rain in recent decades is undeniable (2). Against the background of climate change and the environmental impact of cereal cultivation, there are many arguments for favoring rye in the cultivation of feed grains for pig fattening. Nonetheless, higher energy and protein yields of wheat and corn per ha (3, 4) and certain reservations about higher dietary rye levels for pigs have caused this traditional cereal to disappear in diets for the most part (4, 5). However, the breeding of hybrid lines has resulted in significantly increased yields without forcing the risk of ergot contamination (4). In comparison to wheat, commonly used in diets for pigs, rye is more efficient in using limited resources like water and phosphorus (6). Among other things, this is why cultivation generates a relatively low CO_2_ footprint (7). Thus, an old and traditional feedstuff is experiencing growing interest (8–15). Generally, wheat and rye are characterized by different amounts of starch and crude fiber (3), moreover crude protein levels of wheat are 17–33% higher than those of rye (3, 16). Nevertheless, it is worth mentioning that rye has the more lysine-rich aminogram (3, 17, 18). The ileal digestibility of crude protein is generally lower for rye in comparison to wheat (16, 18, 19). The sugar content of rye is about twice as high as in wheat. With regard to the other crude nutrients and also energy density, there are no major differences (16). Moreover, rye has the highest phytase activity, thus favoring phosphorus utilization (20). In addition, rye has various digestive favorable physiological effects in animals and humans, especially a very high dietary fiber content. Marked differences arise in the content of non-starch polysaccharides (NSP). Rye contains 1.3 to 1.4 times more NSP than wheat (3, 21, 22). A large variance is found in the specification of the fructan content, while the values given for rye are generally up to three times higher than those for wheat (3, 18, 22, 23). Furthermore, the proportion of water-soluble arabinoxylans in rye is significantly higher than in all other cereals (4, 23). Rye-based diets result in significantly higher extract viscosity within the digesta of the stomach and small intestine, which are likely to influence digestibility (12). Nevertheless, higher concentrations of lactic acid and short chain fatty acids due to an intensified fermentation in the digesta could positively influence animal health. A recent experimental study indicates that favorable effects against zoonotically relevant pathogens in pig herds (salmonella) of rye-rich compound feeds might occur. It was shown by Chuppava et al. (24) that salmonella excretion from artificially infected pigs was significantly reduced when high rye levels (69%) were used instead of wheat.

To use rye as a resource-efficient alternative to wheat, and thus also to be able to use its positive effects on animal health, it is necessary to critically test whether, and to what extent, feed intake, digestibility, performance, including feed conversion ratio (FCR), and intestinal conditions as well as fecal characteristics might be influenced when rye is used instead of wheat. Young fattening pigs were deliberately selected for this study, in which disturbances in the digestive tract are very rapidly accompanied by changes in fecal condition.

## 2. Materials and methods

### 2.1. Ethical statement

The experiments were carried out in accordance with German regulations. The studies involving animals were reviewed and approved by the Animal Welfare Officer of the University of Veterinary Medicine Hannover, Hannover, Germany (reference: TiHo-T-2018-24).

### 2.2. Animals and housing

A total of 40 piglets, owned by the Institute for Animal Nutrition, University of Veterinary Medicine Hannover, were housed individually during 5 weeks in the stables of the Institute. The weaned piglets of cross genetics (BHZP GmbH, Dahlenburg-Ellringen, Germany; db. Victoria ♀ × db. 77 ♂, Norwegian Landrace ♀ × German Large White ♂) were allocated to four feeding groups. Pigs were selected on the basis of body weight, sex and sow (siblings distributed between groups) to form four comparable treatment groups. The animals were housed individually in 1 m × 3 m pens with concrete floors, equipped with enrichment materials and nipple drinkers, which provide drinking water *ad libitum* at any time. Visual and tactile contact between the animals was possible through a hole installed in the partitioning walls between the pens. At the beginning of the feeding trial, the piglets were 46.8 ± 5.28 days old with an average bodyweight of 16.1 ± 4.13 kg.

### 2.3. Diets

Each group was fed a pelleted diet consisting of wheat and/or rye, barley, soy, potato protein, and a mineral supplement. The sum of wheat and rye was 69% in all diets, whereby the compound feed of each group was characterized by a different ratio of wheat to rye (Table 1). Diet I, which contained only wheat can be considered as control.

**Table 1:** Composition of the diets (%).

Diet	I	II	III	IV
Group	3/3 wheat	1/3 rye	2/3 rye	3/3 rye
Ingredients				
Wheat	69.0	46.0	23.0	0
Rye	0	23.0	46.0	69.0
Soybean meal^*^	11.5	11.5	11.5	11.5
Barley	10.0	10.0	10.0	10.0
Potato protein	5.10	4.95	4.90	4.90
Calcium carbonate	1.00	1.00	0.95	0.90
Monocalcium phosphate	0.90	0.90	0.95	1.00
Fat (soybean oil)	0.50	0.50	0.50	0.50
Sodium chloride	0.35	0.40	0.40	0.40
Feed additives^**^	1.65	1.75	1.80	1.80

*Extracted soybean meal (steam heated with soapstock) made from genetically modified soybeans. **Additives (per kg as fed); feed additives: vitamin A (12,000 IU), vitamin D3 (2000 IU), vitamin E (150 mg), copper from copper-(II)-glycinate chelate hydrate (4 mg), copper from copper-(II)-sulfate pentahydrate (110 mg), manganese from manganese glycine manganese chelate hydrate (35 mg), manganese from manganese-(II)-oxide (45 mg), zinc from glycine zinc chelate hydrate (40 mg), zinc from zinc oxide (80 mg), iron from iron-(II)-sulfate monohydrate (200 mg), iodine from calcium iodate anhydrous (2.0 mg), and selenium from sodium selenite (0.40 mg).

The chemical composition as well as the particle size distribution of the pelleted diets are described in Table 2. The content of non-starch-polysaccharides (NSP), arabinoxylans, and dietary fiber were analyzed for diets I and IV only.

**Table 2:** Analyzed nutrient composition of compound feeds (g/kg dry matter [DM]).

Diet	I	II	III	IV
Group	3/3 wheat	1/3 rye	2/3 rye	3/3 rye
DM-content (g/kg as fed)	897	897	894	899
Crude ash	48.4	53.2	46.2	51.3
Crude protein	205	205	198	198
Crude fat	27.4	28.1	32.6	24.5
Crude fiber	26.2	24.9	29.9	22.0
Nitrogen-free extracts	625	622	624	637
Starch	530	514	493	491
Sugar	41.3	46.5	52.1	60.0
Lysine	14.7	14.7	14.4	15.0
Methionine	5.24	5.22	4.64	5.60
Cystine	3.97	3.56	3.54	3.34
ME (MJ/kg DM)^1^	15.8	15.8	15.7	15.7
NSP (total)	123	–	–	140
NSP (insoluble)	88	–	–	93
Arabinoxylans (total)	63	–	–	74
Arabinoxylans (soluble)	18	–	–	27
Dietary fiber	143	–	–	156
Mass of particle with size (%)				
>1 mm	27.5	28.2	23.6	22.4
<0.2 mm	42.0	42.8	41.1	45.4

^1^Calculated from nutrient composition using official calculation method (25).

### 2.4. Experimental design and chemical analysis

In the first week of the study, the piglets were acclimatized to the experimental diets by a gradual changeover from the previously fed diet. After adaptation, the animals were fed 4 weeks with the respective compound feed (CF) variant *ad libitum*. The animals received the pelleted diet every morning at the beginning of an experimental day (08:30). A mass was chosen that also resulted in feed residuals on the following day. Care was taken to ensure that no pellets remained in the trough or pen from the previous day. The mass that was not ingested was weighed in the morning before feeding. The difference between weighing in and weighing out gave the mass of ingested feed in original substance (as fed). To calculate the actual ingested mass, the backweights of each experimental week were collected individually, pooled, and the dry matter content (DM) was determined in an aliquot. The dry matter contents of the diets were known from the previous analyses.

Body weight (BW) of each animal was determined weekly. The feed conversion ratio (FCR) resulted from the quotient of feed intake (kg as fed) and body mass gain.

The apparent total tract digestibility was determined by collection method (26, 27). In addition, a visual assessment of the fecal quality was conducted using a semi-quantitative score (28) (See Table 3). The feces were collected over 5 days in the second week of the trial. Immediately before the start of the collection phase, the pens were thoroughly cleaned to avoid any contamination of the feces. The fecal collection was staggered by 1 day from feeding, since a 24 h passage time of the digesta was assumed. The collection phase began at the same time as the morning feeding at 8:30 a.m. and ended again after 5 days at the aforementioned time. Collection was done at hourly intervals daily from 7 am to 9 pm. The feces were collected from the ground with a spatula as loss-free as possible and transferred into a sample container. A distinction was made between “non-contaminated” and “contaminated” (urine, feed, etc.) feces. The feces deposited overnight were considered potentially urine contaminated. This and feces deposited by 8:30 counted as the previous day. The collected feces were stored overnight in a refrigerator at 6°C. At the end of the experimental day, the feces were weighed individually and separately (contaminated/non-contaminated) (BP 1200, Fa. Sartorius AG, Göttingen) and frozen at −18°C. After completion of the collection week, thawing and individual mixing of the feces (contaminated and non-contaminated separately) was performed. The homogenization of the pool sample was done with a drill equipped with a stirring attachment (PSB 750 RCE, Fa. Bosch, Stuttgart). An aliquot was then taken from the pool sample and the dry matter content was determined. This was followed by freeze-drying of the aliquots from the non-contaminated pool samples.

**Table 3:** Score for the assessment of fecal quality according to Borgelt (28).

Score	Consistency
1	firm, formed
2	pulpy, formed
3	pulpy, unformed
4	soupy
5	watery

At the end of an experimental day, the feces were homogenized and weighed individually to receive a daily fecal sample for every pig to determine the DM content. The remaining fecal samples were frozen at −18°C. After the end of the collection period, the faecal samples were defrosted and homogenized individually for every piglet. The apparent total tract digestibility (ATTD) was calculated according to the following formula (25):


$$\mathrm{ATTD}\left(\%\right)=\left(\frac{\mathrm{nutrient amount in feed}-\mathrm{nutrient amount in feces}}{\mathrm{nutrient amount in feed}}\right)\times 100$$

The samples of feed, digesta, and feces were analyzed by standard procedures in accordance with the official methods of the Agriculture and Testing Research Institutes (VDLUFA) (29). Feed particle size distribution was assessed by the wet-sieve method in accordance with Wolf et al. (30).

### 2.5. Dissections

Anesthesia was conducted using Ketamidor^®^ (100 mg/mL, Richter Pharma, Wels, Austria; active ingredient: ketamine hydrochloride, dosage: 20 mg/kg i.m.) and Stresnil^®^ (40 mg/mL, Lilly Deutschland GmbH, Bad Homburg, Germany; active ingredient: azaperone, dosage: 2 mg/kg i.m.). After the neuroleptanalgesia had set in, the pigs were euthanized by intracardiac injection of T61^®^ (Intervet Deutschland GmbH, Unterschleissheim, Germany; active ingredients: tetracaine hydrochloride, mebezonium iodide, embutramide, dosage: 0.3 mL/kg i.c.). The dissection started with opening the pigs from the pelvis along the linea alba to the sternum. The stomach was ligated both towards the esophagus and the duodenum with double ligatures. After another double ligature was placed in front of the rectum, the intestinal tract was exenterated *in toto*. The small intestine, cecum, and colon were then separated by further double ligatures. The organs were separated and weighed. Using the thumb and forefinger, the contents were spread out under continuous well dosed pressure. After this careful emptying, the organs were weighed again to determine the weight of the organs and the mass of the digesta.

### 2.6. Statistical analyses

The analysis of available data was performed using the computer programs Statistical Analyses System for Windows, SAS^®^ 9.4 by means of Enterprise Guide Client Version 7.1 (SAS Institute Inc., Cary, NC, United States). Descriptive statistics included calculating measures such as mean, standard deviation, and percentages. A test for normal distribution was performed using distribution analysis by means of the Shapiro–Wilk test for analytical evaluation. Depending on this, parametric and non-parametric procedures were then used. Normally distributed model residuals were tested by analysis of variance or using a multiple spanning test (Ryan-Einot-Gabriel-Welsch-Q test). For non-normally distributed data or values in the form of a score, the Kruskal–Wallis test was applied accordingly. The significance level was determined as alpha = 5% (*p* < 0.05). Significant differences were indicated by appending different superscript letters (a,b,c, …).

## 3. Results

### 3.1. Performance parameters

The average daily feed intake (ADFI) was recorded daily and is shown in Table 4. With an increasing proportion of rye in the diet, no adverse effects could be determined. Moreover no systemic effect on weight gains was found. From the feed intake and the weight gains of the animals, the feed conversion ratio was calculated. With a higher dietary rye level, the feed conversion ratio increased.

**Table 4:** Average daily feed intake (ADFI, mean ± SD), average daily weight gain (ADWG, mean ± SD), and feed conversion ratio (FCR, mean ± SD).

	3/3 wheat	1/3 rye	2/3 rye	3/3 rye	Value of p*
ADFI g/d	1,378 ± 94.9	1,337 ± 89.0	1,399 ± 186	1,401 ± 179	0.743
ADWG g/d	883 ± 68.9	862 ± 59.5	865 ± 104	839 ± 78.1	0.676
FCR kg/kg**	1.56 ± 0.063^b^	1.55 ± 0.077^b^	1.62 ± 0.089^a,b^	1.67 ± 0.093^a^	**0.013**

*Statistics: REGWQ; ^**^ kg feed (as fed)/body weight gain (kg); ^a,b^, indicates significant differences between groups.

### 3.2. Apparent total tract digestibility

During the second week, the apparent total tract digestibility (ATTD) was determined. Significant differences were found in the ATTD of ether extract (EE) only (Table 5).

**Table 5:** Apparent total tract digestibility (ATTD, %) of organic matter and further crude nutrients (mean ± SD).

ATTD of	3/3 wheat	1/3 rye	2/3 rye	3/3 rye	Value of p*
OM	87.8 ± 3.09	87.7 ± 2.02	86.1 ± 1.33	87.6 ± 2.18	0.335
CP	81.6 ± 5.96	80.4 ± 5.34	77.7 ± 3.20	77.4 ± 5.12	0.064
EE	65.9 ± 7.75^a^	63.1 ± 8.73^a^	67.7 ± 3.62^a^	53.7 ± 9.30^b^	**0.001**
CF	26.5 ± 18.4	30.0 ± 14.1	33.0 ± 6.10	35.2 ± 12.0	0.502
NfE	92.4 ± 1.97	92.6 ± 1.43	91.4 ± 1.09	92.5 ± 1.34	0.136

^a,b,c^ indicates significant differences between groups; ^*^Statistics: REGWQ/Kruskal–Wallis test.

### 3.3. Fecal characteristics

There were no significant differences in fecal consistency, dry matter content, and pH values (Table 6).

**Table 6:** Score, dry matter content (DM; g/kg), and pH of feces (mean ± SD).

	3/3 wheat	1/3 rye	2/3 rye	3/3 rye	Value of p*
Score	1.98 ± 0.877	1.94 ± 0.660	2.18 ± 0.851	1.90 ± 0.662	0.853
DM g/kg	250.1 ± 28.2	269.6 ± 39.6	239.8 ± 41.0	261.4 ± 28.5	0.260
pH	6.28 ± 0.309	6.33 ± 0.370	6.17 ± 0.483	6.21 ± 0.456	0.809

^*^Statistics: REGWQ/Kruskal–Wallis test.

### 3.4. Organ and digesta mass and products of bacterial fermentation

No significant differences in organ and digesta mass of cecum and colon, concentrations of SCFA, and lactic acid were found, partly due to high standard deviations of the values.

Nevertheless, numerically differences occur. For example, numerically higher masses of the emptied colon and colon digesta were found with highest dietary rye levels. In addition, increasing rye contents in the compound feed resulted in numerically higher (not significant) lactic acid concentrations in the content of the cecum and colon (Table 7).

**Table 7:** Concentrations (mmol/kg DM) of SCFA and lactic acid in the content of cecum and colon (mean ± SD).

		3/3 wheat	1/3 rye	2/3 rye	3/3 rye	Value of *p*
Acetic acid	Cecum	693 ± 163	790 ± 270	659 ± 151	763 ± 354	0.614
	Colon	410 ± 164	422 ± 123	385 ± 101	421 ± 200	0.979
Propionic acid	Cecum	515 ± 164	495 ± 162	507 ± 100	499 ± 152	0.949
	Colon	239 ± 116	216 ± 65.8	242 ± 105	266 ± 151	0.933
i-butyric acid	Cecum	2.95 ± 2.71	3.70 ± 4.09	2.99 ± 1.40	4.12 ± 3.41	0.792
	Colon	7.78 ± 3.44	8.05 ± 2.01	7.83 ± 2.63	6.75 ± 3.88	0.660
n-butyric acid	Cecum	154 ± 76.7	207 ± 83.2	183 ± 85.0	186 ± 82.1	0.619
	Colon	116 ± 46.3	121 ± 47.4	119 ± 61.5	129 ± 69.5	0.989
i-valeric acid	Cecum	3.52 ± 2.46	4.47 ± 3.94	4.41 ± 3.43	7.97 ± 8.34	0.783
	Colon	11.0 ± 4.49	11.0 ± 3.89	11.0 ± 4.46	8.44 ± 4.08	0.356
n-valeric acid	Cecum	38.5 ± 18.3	52.9 ± 29.7	54.3 ± 37.5	47.6 ± 24.4	0.760
	Colon	34.5 ± 14.1	37.0 ± 21.5	47.7 ± 28.5	38.6 ± 19.4	0.802
L-lactate	Cecum	133 ± 185	171 ± 166	162 ± 210	332 ± 335	0.466
	Colon	25.5 ± 61.2	11.4 ± 33.7	25.8 ± 49.0	80.7 ± 152	0.466
D-lactate	Cecum	47.1 ± 63.4	59.7 ± 66.3	56.3 ± 56.5	113 ± 100	0.404
	Colon	9.69 ± 20.1	4.20 ± 11.2	9.74 ± 18.1	34.4 ± 62.1	0.430

*Statistics: REGWQ/Kruskal–Wallis test.

## 4. Discussion

When wheat was partially to completely replaced by rye, all groups of pigs achieved daily ADFI of more than 1,300 g. According to Thacker et al. (31), a proportion of 60% either low or high viscosity population rye in the diet for fattening pigs (BW: 17–34 kg) resulted in a significantly lower feed intake of about 25% (high viscosity rye) respectively 17% (low viscosity rye) compared to the control group fed a barley-based diet. Grela and Walkiewicz (32) described a significantly lower feed intake when a diet with 20% of population rye (pre-fattening period) or 40% of rye (finishing period) was fed. In contrast, the use of 69% population rye compared to the use of a diet with 69% wheat had no negative influence on the feed intake of pigs from the 35th until 56th day of life (33). According to Weber et al. (34), higher feed intakes were achieved when a diet with proportions of 15–30% population rye were fed to piglets (BW: 10–30 kg) compared to a diet based on wheat and barley. In addition, a current study also with hybrid rye showed no negative effect of replacing 48% wheat with 48% rye in diets for weaning pigs (BW: 8–22 kg) (35). Under an artificial infection with Salmonella in young fattening pigs (BW: 10–28 kg), equal feed intake values were also obtained when 69% wheat was replaced with 69% hybrid rye (24). Trials with fattening pigs fed increasing levels of rye instead of wheat throughout the fattening phase showed reduced feed intakes and daily gains (*p* < 0.05), even though the weight of the animals did not differ. At very high input rates, the addition of enzymes had a positive effect on FCR (36). Studies in which weaned piglets were fed increasing proportions of hybrid rye instead of maize showed that rye had a favorable effect on gains at the beginning of the study (up to day 7 after weaning), and in the further phases feed intake in particular was favorably influenced. However, since the gains remained the same, an increasing effect on the FCR was also described (37). Direct comparison in pig fattening of barley, triticale and rye showed no difference in terms of feed intake and FCR. Nevertheless, daily gains were higher when fed triticale, compared to rye, and thus the final weight of the animals was also higher. The results of barley were in between (9). Generally, these study show that changes in feed intake can occur with the use of rye, but this is not always the case. In general, however, an increase in feed intake is not expected when wheat is replaced. In previous recommendations, negative effects on feed intake due to lower palatability of rye were mentioned as a reason for limitations for the use of rye in pig feeding (4, 34). According to Grela and Walkiewicz (32), tannins and pentosans as well as alkylresorcinols are anti-nutritional substances of rye. Thus, lower feed intake was observed in pigs when rye oil with a correspondingly high alkylresorcinol content was added to the diet (38). Generally, due to deviations in the analytical method and apparently varying contents, given values for wheat and rye vary considerably (39–42). According to recent studies, rye (720–1,284 mg/kg) and wheat (138–763 mg/kg) reach similarly high alkylresorcinol contents in contrast to older studies (43–46).

With increasing rye contents in the diet, no significant differences regarding the ATTD of CP were found. Although with a total replacement numerically lower values for the ATTD of CP occurred. A lower apparent ileal digestibility of crude protein, in comparison to wheat, found by Ellner et al. (35) might result from higher extract viscosity in the digesta or from so-called doughballs in gastric digesta found by Wilke et al. 2021 (47). With regard to ATTD of OM, there were no significant differences when wheat was completely replaced by rye in the diet. Nevertheless, significant differences in the prececal digestibility between the two cereals can be assumed (18, 35, 64). According to Hartung et al. (48), the diet with an amount of 69% of rye compared to the control (69% wheat) showed a significantly lower prececal digestibility of the OM, whereas, as in the present study, there were no differences in ATTD. This might be explained by the significantly higher proportion of NSP (3, 48), which can only be microbially degraded in the large intestine, which in turn can even lead to an increase in the concentration of short-chain fatty acids (e.g., butyrate) in the blood (49). Generally, a lower prececal digestibility of the OM leads to a higher influx of fermentable substances in the hind gut, resulting in a higher level of gut fill (50). Differences in the digestibility of EE might also occur as a result of a higher amount of fermentable fiber in the diet, as it was shown in previous investigations (51). This may result from general effects on macronutrient digestibility from higher fiber content (52), also conceivable, when there is an increased influx of fermentable substances in the hindgut, additional fats are synthesized by the microorganisms, which thus could lead to an apparently lower digestibility of crude fat (48). Furthermore, the numerically increasing digestibility of crude fiber with higher levels of rye in the diet might indicate that the dietary fiber from rye has a higher digestibility or fermentability compared to wheat. Even though there were no significant differences regarding the products of fermentation in this study, generally an enhanced fermentation in the hindgut can be associated with a longer lasting satiety (50), which can be advantageous in restrictive feeding of pigs in herds (e.g., pregnant sows).

No differences regarding the gains occurred. According to Thacker, Campbell and Scoles (31), the use of 60% rye in the diet resulted in significantly lower daily weight gains in fattening pigs (BW: 17–34 kg). With an increasing proportion of rye (up to 80%) in the diet, lower performance was observed in fattening pigs (BW: 30–70 kg) (32). Contrary, 65.5 and 69% rye in the diets of young pigs (BW: 15–30 kg) compared to a wheat-based diet did not lead to a reduction in weight gains (34). Moreover, there were no negative effects on weight gains when wheat and barley were replaced by rye in the diet (34, 53). According to current studies, the replacement of neither 48% nor 69% wheat with rye reduced ADWG (24, 35).

When wheat was totally replaced by rye in the diet, a significantly higher FCR occurred. In piglets (BW: ~9–30 kg), a higher feed conversion ratio was determined when wheat and small amounts of barley were replaced by rye in the diet and thus a rye proportion of up to 30% was fed. The control group reached a feed conversion ratio of 1.57 kg/kg, whereas in the experimental group (30% rye), an FCR of 1.64 kg/kg was determined (34). It was found that in comparison to wheat, rye-based diets showed a lower prececal digestibility (18, 48). A higher FCR of rye-based diets might result from the lowered prececal digestibility, as a lower proportion of macronutrients is directly and efficiently available in the small intestine. In addition, diets high in dietary fiber are associated with lower energy contents. Even if an increasing amount of SCFA is available in the colon (energy source) with higher amounts of dietary fiber, the dietary lower energy content of these diets cannot be compensated (54).

The parameter of fecal characteristics is important, as it determines cleanliness of the stable to a large extent. The fecal characteristics were examined visually and chemically using laboratory analysis (DM content and pH-value). The evaluated score values (1.9–2.2, Table 6) corresponded to a physiological fecal consistency, which is described as “pulpy, formed” (28). There were no significant differences in the DM content of the feces. Numerically lower pH values in the feces with rye-based diets might indicate an increased fermentation in the hind gut (53). Generally, fermentation in the large intestine is in turn determined by the influx of substances not digested in the anterior intestinal tract, e.g., NSP or resistant starch (55, 56). The measured values for the products from fermentation varied to a large extent (i.e., high standard deviation for lactate) (See Tables 7 8). Nevertheless, with increasing proportions of rye in the diet, numerically higher concentrations of lactate were measured in the group fed a diet based on 69% of rye, compared to the control. In this context, it must be borne in mind that the production rates can only be inferred to a limited extent from the concentrations, since the intestine can quickly absorb high amounts, especially of SCFA (57). With this, higher levels of butyrate within the animals can then also be expected systemically as a result of the intake of diets, rich in fermentable fiber (49). With a forced formation of butyric acid in the hind gut, favorable effects against entering Salmonella can be assumed (58–63).

**Table 8:** Mass of organ (g) and digesta (g, dry matter) of cecum and colon (mean ± SD).

		3/3 wheat	1/3 rye	2/3 rye	3/3 rye	Value of *p*
Organ mass	Cecum	87.3 ± 19.4	79.6 ± 21.8	90.9 ± 25.8	84.9 ± 14.1	0.673
	Colon	646 ± 114	680 ± 135	681 ± 117	769 ± 89.3	0.120
Digesta mass	Cecum	19.2 ± 13.3	23.9 ± 21.2	18.0 ± 5.23	17.1 ± 10.7	0.613
	Colon	120 ± 48.0	145 ± 52.1	130 ± 56.9	152 ± 53.6	0.534

*Statistics: REGWQ/Kruskal–Wallis test.

## 5. Conclusion

Even with really high dietary levels of rye (69%), the young fattening pigs achieved an average daily feed intake of more than 1,300 g DM. Digestibility rates (%) of organic matter and crude protein did not differ. With increasing amounts of rye in the diet, digestibility of EE was reduced. Daily weight gains were not significantly affected. When wheat was replaced completely by rye, the FCR increased. Further experiments are needed to determine not only the concentrations but also the production rates of various favorable products of the microbiota. Nevertheless, no negative effects (feed intake, performance, and fecal characteristics) of feeding even high proportions of hybrid rye in young fattening pigs were found.

## Data availability statement

The raw data supporting the conclusions of this article will be made available by the authors, without undue reservation.

## Ethics statement

The experiments were carried out in accordance with German regulations. The studies involving animals were reviewed and approved by the Animal Welfare Officer of the University of Veterinary Medicine Hannover, Hannover, Germany (reference: TiHo-T-2018-24).

## Author contributions

VW and JK contributed to the conception, design of the study, and wrote sections of the manuscript. VW organized the database, performed the statistical analysis, and wrote the first draft of the manuscript. All authors contributed to the article and approved the submitted version.

## Funding

The project was supported by funds of the Federal Ministry of Food and Agriculture (BMEL) based on a decision of the Parliament of the Federal Republic of Germany via the Federal Office for Agriculture and Food (BLE) under the innovation support program (FKZ: 281B101016). This Open Access publication was funded by the Deutsche Forschungsgemeinschaft (DFG, German Research Foundation)—491094227 “Open Access Publication Funding” and the University of Veterinary Medicine Hannover, Foundation.

## Conflict of interest

The authors declare that the research was conducted in the absence of any commercial or financial relationships that could be construed as a potential conflict of interest.

## Publisher’s note

All claims expressed in this article are solely those of the authors and do not necessarily represent those of their affiliated organizations, or those of the publisher, the editors and the reviewers. Any product that may be evaluated in this article, or claim that may be made by its manufacturer, is not guaranteed or endorsed by the publisher.
